# Latent Tuberculosis among Persons at Risk for Infection with HIV, Tijuana, Mexico

**DOI:** 10.3201/eid1605.091446

**Published:** 2010-05

**Authors:** Richard S. Garfein, Rafael Laniado-Laborin, Timothy C. Rodwell, Remedios Lozada, Robert Deiss, Jose Luis Burgos, Jazmine Cuevas-Mota, Paris Cerecer, Kathleen Moser, Maria Luisa Volker, Steffanie A. Strathdee

**Affiliations:** University of California San Diego School of Medicine, San Diego, California, USA (R.S. Garfein, T.C. Rodwell, R. Deiss, J.L. Burgos, J. Cuevas-Mota, S.A. Strathdee); Universidad Autonoma de Baja California, Tijuana, Mexico (R. Laniado-Laborin); Patronato Pro-Comité Municipal de Prevención del SIDA, Tijuana (R. Lozada); Instituto de Servicios de Salud Pública del Estado de Baja California, Tijuana (P. Cerecer, M.L. Volker); San Diego County Department of Health and Human Services, San Diego (K. Moser)

**Keywords:** Tuberculosis, Mycobacterium tuberculosis, substance abuse, HIV, injection drug use, commercial sex worker, Mexico, screening, epidemiology, research

## Abstract

Because there is little routine tuberculosis (TB) screening in Mexico, the prevalence of latent TB infection (LTBI) is unknown. In the context of an increasing HIV epidemic in Tijuana, Mexico, understanding prevalence of LTBI to anticipate emergence of increased LTBI reactivation is critical. Therefore, we recruited injection drug users, noninjection drug users, female sex workers, and homeless persons for a study involving risk assessment, rapid HIV testing, and TB screening. Of 503 participants, the overall prevalences of TB infection, HIV infection, and TB/HIV co-infection were 57%, 4.2%, and 2.2%, respectively; no significant differences by risk group (p>0.05) were observed. Two participants had TB (prevalence 398/100,000). Incarceration in Mexico (odds ratio [OR] 2.28), age (OR 1.03 per year), and years lived in Tijuana (OR 1.02 per year) were independently associated with TB infection (p<0.05). Frequent LTBI in marginalized persons may lead to increases in TB as HIV spreads.

## Abstract 2

Interventions that prevent HIV transmission and TB reactivation are needed.

Tuberculosis (TB) is endemic to Mexico. The national TB incidence is estimated to be 16.2 cases/100,000, but regional rates, particularly along the United States–Mexico border are much higher (*1*). Baja California, which shares a border with California, has the highest incidence of pulmonary TB in Mexico (57 cases/100,000), which is >3× the national average (*2*). Furthermore, California and Baja California have the highest incidence rates of all the border states in their respective countries (*1*). Transmission of TB from high-prevalence countries to low-prevalence countries, such as the United States, poses a major public health concern. Tijuana, the largest city in Baja California, Mexico, lies ≈20 miles south of downtown San Diego, California, and these 2 cities are loosely separated by the busiest land border crossing in the world, with >90,000 passenger vehicles crossing in both directions daily (*3*). As a possible consequence of this close binational association, San Diego has a slightly higher incidence of TB than California (8.4 per 100,000 and 7.0 per 100,000, respectively) (*4*,*5*).

Otherwise healthy persons with dormant or latent tuberculosis infection (LTBI) have a 10% lifetime risk that dormant mycobacteria will become active and cause TB. Persons co-infected with HIV and LTBI have a risk for TB reactivation of 10% per year (*6*). Consequently, TB is a leading cause of death worldwide among persons co-infected with TB and HIV (*7*). Although HIV prevalence in Mexico is lower than that in the United States, Tijuana is currently experiencing an emerging HIV epidemic (*8*), with increasing prevalence observed among high-risk groups such as injection drug users (IDUs) and female sex workers (FSWs) (*9*–*11*). The potential for rapid HIV transmission among IDUs in Mexico is highlighted by findings from Tijuana and Ciudad Juarez (a border city south of El Paso, Texas), which showed that 95% of IDUs in these cities had antibodies against hepatitis C virus (a marker of unsafe injection practices), and most IDUs had self-reported risk factors for sexual or parenteral exposure to HIV (*12*). Thus, there is concern that increased HIV incidence would result in LTBI reactivation among IDUs who spread active TB to other populations, which would lead to a more generalized epidemic.

Homeless persons and noninjecting drug users (NIDUs) are also at increased risk for co-infection with TB/HIV. In low-prevalence countries, these risk groups have higher rates of LTBI than those in the general population (*13*–*15*). Although estimates of LTBI and their correlates among FSWs are not well documented, an increasing percentage of FSWs in Tijuana are infected with HIV and report multiple potential risk factors for TB, including injection drug use and sexual contact with IDUs (*10*). Thus, the need for accurate estimates of LTBI prevalence in high-risk marginalized populations is clear.

In the United States, LTBI screening and prophylactic treatment have played a major role in reducing co-infection with TB/HIV in most regions and communities. However, LTBI screening, which is usually conducted by using the tuberculin skin test (TST), is uncommon in countries such as Mexico, which still uses *Mycobacterium bovis* BCG vaccination universally, because the TST has reduced specificity in vaccinated persons (*16*). Whole-blood interferon-γ release assays (IGRAs), which measure cellular immune response to purified proteins found in *M*. *tuberculosis,* but not in BCG vaccine strains, provide a means for more accurately estimating TB infection prevalence in countries such as Mexico (*17*). A study that measured TB infection prevalence in Mexico by using an IGRA found that 67% of IDUs in Tijuana were positive (*18*). However, that study did not differentiate between LTBI and active TB and included only IDUs. The purposes of the current study, known as PreveTB, were to measure the prevalence of TB and HIV among marginalized populations of Tijuana who are at a high risk for becoming co-infected with HIV and TB, estimate the prevalence of active TB in this group, and identify correlates of LTBI.

## Methods

### Study Population

Participants were recruited in Tijuana, Mexico, during April 2007–July 2007, by street outreach, targeted advertising, and word-of-mouth. Persons were eligible to participate if they were >18 years of age, provided informed consent, planned on staying in Tijuana for the next 30 days, and reported at least 1 of the following characteristics in the 6 months before enrollment: use of noninjected illicit drugs other than marijuana, injection drug use, receipt of money/goods in exchange for sex, and homelessness or unstable housing. Unstable housing was defined as living primarily in a rented hotel room, migrant work camp, or medical/drug treatment facility.

### Data Collection

Participant interviews were conducted and biological samples were obtained at PrevenCasa, a community-based harm-reduction and research facility located in the Zona Norte neighborhood of Tijuana, which abuts the commercial sex trade district. Computer-assisted personal interviewing technology (QDS; Nova Research Company, Bethesda, MD, USA) was used to facilitate participant interviews. Interviews were conducted in Spanish by trained Mexican interviewers experienced with the specific populations. Questions included sociodemographic characteristics, putative risk factors for TB and HIV infection, TB knowledge and exposure history, and presence of TB-related symptoms. The survey instrument was developed in English, translated into Spanish, and then back-translated into English to verify accuracy and meaning. Monetary reimbursement of US $20 was offered to participants to compensate them for time and transportation.

During the computer-assisted personal interview, persons who reported a persistent cough for >3 weeks and an additional symptom indicative of TB (fever or chills, night sweats, swollen lymph nodes, hoarseness, shortness of breath, joint pain, fatigue, and unexplained weight loss) or hemoptysis with or without other symptoms, were considered suspected active TB case-patients. After the interview, these persons were asked to provide 3 sputum samples for acid-fast bacilli (AFB) smears; the first of these samples was collected immediately. The remaining 2 samples were collected on subsequent days. These participants were also transported to a nearby radiologic center for chest radiography to identify evidence of pathologic changes consistent with TB. Incentives of US $5 were given for each additional sputum collection visit and upon completion of a chest radiograph, as recommended by FitzGerald et al. (*19*). Participants with >1 AFB-positive sputum smear or chest radiography findings consistent with TB were determined likely to have active TB and were referred to the central public health clinic (Instituto de Servicios de Salud Pública del Estado de Baja California [ISESALUD]) for clinical confirmation and treatment through the national TB program. The study protocol was reviewed and approved by ethics committees at the University of California, San Diego, and the Tijuana General Hospital.

### Laboratory Testing

*M*. *tuberculosis* infection was detected by using an IGRA (QuantiFERON TB Gold In-Tube [QFT] assay; Cellestis Ltd., Carnegie, Victoria, Australia), an in vitro assay that uses an ELISA to detect interferon-γ released by whole blood samples after introduction of 6-kDa early secretory antigenic target protein, culture filtrate protein 10, and TB7.7 protein, which mimic antigens specific to the *M*. *tuberculosis* complex present in patients with TB and LTBI (*20*). In 2005, the US Food and Drug Administration (Silver Spring, MD, USA) approved the QFT assay for detection of *M*. *tuberculosis* infection, and the US Centers for Disease Control and Prevention (Atlanta, GA, USA) determined that this assay may be used to detect *M*. *tuberculosis* in all situations in which the TST is used (*17*). Antibodies against HIV were detected by using the Determine Rapid HIV Antibody Test (Abbott Laboratories, Boston, MA, USA) on site. All positive test results were confirmed at the San Diego County Public Health Laboratory by using an ELISA and an immunofluorescent antibody assay.

After data collection, all persons who did not report symptoms consistent with active TB were given an appointment 4 weeks later to receive the results of the HIV test and QFT assay. At this visit, those who were HIV positive were referred to ISESALUD for medical evaluation, and those who were IGRA positive were offered chest radiography when they returned to obtain results that ruled out active TB. Persons who had active TB by AFB smear or radiography were referred to ISESALUD. Persons who were IGRA positive, but had an unremarkable chest radiograph, were determined to have LTBI.

### Statistical Analysis

Descriptive statistics were used to characterize participants by IGRA status. Associations between participant characteristics and TB infection were evaluated by using the Wilcoxon rank-sum test or Fisher exact test, followed by univariate and multivariate logistic regressions. Variables with p values <0.10 in univariate models were considered for inclusion in multivariate analysis. A backward stepwise model that selected for main effects in the final model was used. At each step, likelihood ratio testing was used to compare nested models until only variables with p values <0.05 remained in the final model.

## Results

### Prevalence of HIV and TB Infection

A total of 527 persons were recruited, of whom 503 met eligibility criteria and had complete HIV and IGRA results. Overall, 57% were positive for TB infection by QFT assay (IGRA+), 4.2% were HIV+ by HIV test, and 2.2% were positive by both tests. Fifty-nine percent of participants were men, median age was 36 years (interquartile range 29–42 years), and median length of time participants had lived in Tijuana was 6 years (interquartile range 1.0–13.3 years). On the basis of nonmutually exclusive groupings, there were 232 (46%) IDUs, 311 (62%) NIDUs, 115 (23%) FSWs, and 280 (56%) homeless persons. The prevalence of IGRA+ results was 63% among IDUs, 58% among NIDUs, 49% among FSWs, and 52% among homeless participants. A total of 14 (2.8%) participants reported symptoms suggestive of active TB, of which 2 persons were diagnosed with active TB by AFB+ smears (prevalence 398/100,000). Because chest radiography was used after recruitment began and was offered at the results visit, only 79 of the 286 IGRA+ participants had chest radiography, of which 8 (10%) had signs of current or past pulmonary TB.

### Correlates of TB Infection

Univariate analysis identified several factors associated with TB infection (Table 1). When compared with IGRA– participants, IGRA+ participants were significantly older (median age 38 vs. 34 years; p<0.01), had lived in Tijuana longer (median 9.0 vs. 3.5 years; p<0.01), were more likely to be men (64.7% vs. 51.6%; p<0.05), had stable sleeping arrangements in the 6 months before data collection (49.3% vs. 37.8%; p<0.05), and had known someone with TB (46.2% vs. 35.9%; p<0.05). When compared with those not in each risk group, IDUs were more likely to be IGRA+, and FSWs and homeless participants were less likely to be IGRA+; no association was found for NIDUs. History of incarceration was associated with TB infection. Persons who had been incarcerated in Mexico were more likely to be IGRA+ (68%) than those incarcerated in the United States (55%) or both countries (62%). To maximize statistical power, in subsequent analyses we combined incarceration in Mexico and the United States into 1 incarceration category. When compared with IGRA– participants, IGRA+ participants were no more likely to report sexually transmitted infections (2.5% vs. 2.3%; p = 0.91) or be HIV+ (3.8% vs. 4.6%; p = 0.67). TB infection was not associated with any other drug administration practice or sexual behavior examined in this study.

**Table 1:** Univariate analysis of factors associated with tuberculosis infection status among high-risk groups for HIV in Tijuana, Mexico, April–July 2007*

Characteristic	Total (n = 503)	IGRA– (n = 217)	IGRA+ (n = 286)	Odds ratio (95% CI)
Risk factor†‡				
Injected drugs	232 (46.1)	86 (39.6)	146 (51.1)	1.59 (1.11–2.27)
Used drugs but never injected	207 (41.2)	94 (43.3)	113 (39.5)	0.86 (0.60–1.22)
Sex work	115 (22.9)	59 (27.2)	56 (19.6)	0.65 (0.43-0.99)
Homeless/unstably housed	280 (55.7)	135 (62.2)	145 (50.7)	0.63 (0.44–0.90)
Median age, y (IQR)	36.0 (29–42)	34.0 (28–40)	38.0 (31–43)	1.04 (1.02–1.06)
Sex				
M	297 (59.0)	112 (51.6)	185 (64.7)	1.00
F	193 (38.4)	99 (45.6)	94 (32.9)	0.58 (0.40–0.83)
Transgender	13 (2.6)	6 (2.8)	7 (2.5)	0.71 (0.23–2.16)
Years lived in Tijuana, median (IQR)	6.04 (1–13.3)	3.51 (0.3–10.0)	9.0 (3.0–16.4)	1.03 (1.012–1.04)
Most frequent type of sleeping arrangement‡				
Stable	223 (44.3)	82 (37.8)	141 (49.3)	1.00
Unstable housing	209 (41.6)	105 (48.4)	104 (36.4)	0.58 (0.39–0.85)
Homeless	71 (14.1)	30 (13.8)	41 (14.3)	0.80 (0.46–1.37)
Ever incarcerated	338 (67.2)	128 (59.0)	210 (73.4)	1.92 (1.32–2.80)
Incarceration status				
Never jailed	165 (32.8)	89 (41.0)	76 (26.6)	1.00
Jailed in USA only	129 (25.6)	58 (26.7)	71(24.8)	1.43 (0.90–2.28)
Jailed in Mexico or Mexico and USA	209 (41.5)	70 (32.3)	139 (48.6)	2.33 (1.53–3.54)
Noninjection drug used most often‡				
None	157 (31.2)	67 (30.9)	90 (31.5)	1.00
Methamphetamines	218 (43.3)	101 (46.5)	117 (40.9)	0.86 (0.57–1.30)
Heroin	52 (10.3)	14 (6.5)	38 (13.3)	2.02 (1.01–4.03)
Any other drug	76 (15.1)	35 (16.1)	41 (14.3)	0.87 (0.50–1.51)
Ever injected illegal drugs	248 (49.3)	92 (42.4)	156 (54.6)	1.63 (1.14–2.33)
Mean (SD) years injected drugs among IDUs	13.5 (13.0)	13.0 (13.5)	14.0 (14.0)	1.02 (0.99–1.05)
Ever known anyone with TB	210 (41.8)	78 (35.9)	132 (46.2)	1.53 (1.06–2.19)

*Values are no. (%) unless otherwise indicated. IGRA, interferon-γ release assay; CI, confidence interval; IQR, interquartile range; IDUs, injection drug users; TB, tuberculosis. †Risk groups are not mutually exclusive; reference group includes all participants without the characteristic. ‡Refers to the past 6 mo.

Multivariate logistic regression analysis (Table 2) showed that TB infection was independently associated with age, increasing years of Tijuana residence, and a history of being incarcerated in Mexico or in Mexico and the United States. For each 1-year increase in age and time lived in Tijuana, participants were 1.03× (95% confidence interval [CI] 1.01×–1.05×) and 1.02× (95% CI 1.01×–1.04×) more likely to be IGRA+, respectively. Persons who had a history of being jailed in Mexico were 2.28× (95% CI 1.48×–3.51×) more likely to be IGRA+ than those who had never been incarcerated.

**Table 2:** Multivariate analysis of factors associated with tuberculosis infection status among high-risk groups for HIV in Tijuana, Mexico, April–July 2007*

Characteristic	Adjusted odds ratio (95% Cl)
Age, y	1.03 (1.01–1.05)
Years lived in Tijuana	1.02 (1.01–1.04)
Incarceration status	
Never jailed	1.00
Jailed in USA only	1.61 (0.98–2.63)
Jailed in Mexico or in Mexico and USA	2.28 (1.48–3.51)

*Odds ratios were adjusted for all other variables. CI, confidence interval.

## Discussion

We found a high prevalence of TB infection among marginalized populations at high risk for HIV infection in Tijuana. Although HIV prevalence in this study (4.2%) was lower than estimates reported among similar populations elsewhere (*21*,*22*), it was higher than that of the general population of Baja California, Mexico (0.8%–0.9%) (*9*,*23*). If HIV prevalence increases among groups who have high LTBI prevalence, reactivation and spread of TB will hamper TB control efforts in the region. Interventions are needed that prevent HIV transmission and LTBI reactivation.

Among IDUs in this study, the TB infection prevalence (63%) was consistent with that in an earlier study of IDUs in Tijuana (*18*), which reported a crude prevalence of 67% and a prevalence of TB infection of 64% after the estimate was adjusted for respondent-driven sampling. This method of sampling of hidden populations enables researchers to adjust prevalence estimates to account for sampling bias (*24*). In addition to confirming the high prevalence of LTBI among IDUs in Tijuana, we found a disturbingly high LTBI prevalence among other hard-to-reach groups. Given that the reference group for associations found between FSWs or homelessness and LTBI status was mostly IDUs, it was not surprising that these factors had odds ratios less than unity. Further studies that include low-risk groups are needed to determine the risk for LTBI among FSWs and homeless persons relative to the general population.

Unlike our earlier study (*18*), in which we referred persons with TB symptoms directly to a community clinic for further evaluation, the current study included AFB smear microscopy and chest radiography to detect active TB before referring participants for care. These modifications enabled us to estimate the prevalence of active TB. Our estimate of TB disease prevalence (398/100,000) was ≈4× higher than the reported TB prevalence for Baja California. Although this estimate is based on a small number of cases, the fact that AFB smears can miss up to 40% of culture-positive TB cases (*25*) indicates that this finding is likely a conservative estimate. Our findings concur with those of other studies, which showed that substance abuse, injection drug use in particular, is associated with increased risk for active TB (*26*).

Age and years of residence in Tijuana were associated with increased odds of TB infection. This finding, along with the fact that TB incidence was estimated to be 57/100,000 in Baja California versus the national rate of 16.2/100,000, suggests that living in this region of Mexico may increase the risk for acquiring *M*. *tuberculosis* infection (*1*). Although having spent time in jail or prison was associated with TB infection in our study, having spent time in a correctional facility in Mexico appeared to be a major risk factor, regardless of also having spent time in a correctional facility in the United States. Transmission of TB is facilitated by close contact with infectious persons, such as those found in correctional facilities, and increased TB incidence rates have been documented during incarceration (*27*,*28*). Inmates at facilities in Mexico may have a higher risk for infection because of the higher prevalence of TB in Mexico than in the United States, the greater densities of inmates in these facilities, and the lack of LTBI screening programs in Mexico. These correctional facilities offer a unique opportunity to reduce TB in the community at large (*29*).

The World Health Organization has estimated that one third of the world’s population has LTBI (*7*), which creates a massive reservoir of disease that is considered a major threat to global TB control (*30*). Persons co-infected with TB and HIV are at greatest risk for reactivation of LTBI; they have an incidence of ≈35–162 cases/1,000 person-years. The second and third highest risks for LTBI reactivation occurs among persons who were recently infected (<12 months earlier) and IDUs, who collectively have an incidence of ≈10–12.9/1,000 person-years, which is independent of their HIV status (*31*). However, in Mexico sufficient resources are not available to treat all with LTBI cases. Therefore, treatment is restricted to those with LTBI cases considered at high risk for reactivation, which is currently defined as children who have had contact with infectious TB case-patients and those co-infected with HIV. As new diagnostic tools capable of identifying LTBI cases at imminent risk for progressing to TB are developed, TB control programs in places such as Tijuana could expand treatment of persons with LTBI in a focused, cost-efficient manner (*32*).

The cross-sectional design of our study precluded us from drawing temporal inferences between TB infection and risk factors we examined. Moreover, information obtained about recent behavior (substance and drug use, sexual behavior, and housing status) may not reflect patterns present at the time participants became infected. This finding could explain why our analysis identified that only lifetime variables were independently associated with TB infection. In addition, this study used convenience sampling methods to enroll participants. Therefore, our results may not be representative of other marginalized populations in Mexico. *M*. *bovis* infections, which accounted for <17% of TB infections in persons born in Mexico who were living in southern California from 2001 through 2005 (*33*), can also cause an IGRA+ result. Thus, some IGRA+ results in this study may have been caused by *M*. *bovis* infections rather than *M*. *tuberculosis* infections. However, we do not believe that this possibility changes our conclusions because TB caused by *M*. *bovis* and TB caused by *M*. *tuberculosis* are essentially indistinguishable clinically (*34*), and both infections cause illness and death in this region (*33*), which are exacerbated by HIV infection.

Our results were based on an IGRA, and although there is no standard procedure for detecting LTBI, studies have consistently reported that IGRAs are more sensitive and specific for detecting active TB than are TSTs (*35*). In addition, 78% of the study participants had a visible BCG scar, which suggested that LTBI would have been overestimated if based on a TST in this population. Although the LTBI estimates in this study suggest treatment specific for high-risk groups, additional studies are needed to obtain comparable estimates from the general population to determine whether interventions should be specific for certain groups or the general population.

Although prevention of infection with HIV should be the top priority for reducing TB risk, the high LTBI prevalence found in this study indicates an unmet need for early TB identification and treatment among populations in Tijuana at risk for HIV infection. TB and HIV screening and treatment can be difficult to accomplish, given that these hard-to-reach populations access medical care infrequently, and drug users and homeless populations have additional barriers to compliance with TB treatment regimens (*36*,*37*). Official guidelines in Mexico currently recommend treatment for LTBI only for exposed children <5 years of age, children 5–14 years of age if they have no history or signs of BCG vaccination, and HIV-infected persons with known exposure to a person with an active case of TB (*38*). However, a separate economic analysis has shown that an LTBI screening program similar to that demonstrated in this study, together with an LTBI treatment strategy, would be cost-effective among IDUs in Tijuana (*39*). Additional studies are needed to determine whether treatment of LTBI would also be cost-effective for the general population and other groups at high risk for TB/HIV co-infection. Expanded TB and HIV screening efforts, coupled with HIV risk reduction interventions and education about TB among these high-risk populations, are needed to avoid reactivation and spread of TB resulting from emergence of HIV in this Mexico/United States border region.
